# The Macrophage-depleting Agent Clodronate Promotes Durable Hematopoietic Chimerism and Donor-specific Skin Allograft Tolerance in Mice

**DOI:** 10.1038/srep22143

**Published:** 2016-02-26

**Authors:** Zhanzhuo Li, Xin Xu, Xingmin Feng, Philip M. Murphy

**Affiliations:** 1 Laboratory of Molecular Immunology, National Institute of Allergy and Infectious Diseases (NIAID), National Institutes of Health, Bethesda, MD, USA; 2 Hematology Branch, National Heart, Lung and Blood Institute (NHLBI), National Institutes of Health, Bethesda, MD, USA

## Abstract

Hematopoietic chimerism is known to promote donor-specific organ allograft tolerance; however, clinical translation has been impeded by the requirement for toxic immunosuppression and large doses of donor bone marrow (BM) cells. Here, we investigated in mice whether durable chimerism might be enhanced by pre-treatment of the recipient with liposomal clodronate, a macrophage depleting agent, with the goal of vacating BM niches for preferential reoccupation by donor hematopoietic stem cells (HSC). We found that liposomal clodronate pretreatment of C57BL/6 mice permitted establishment of durable hematopoietic chimerism when the mice were given a low dose of donor BM cells and transient immunosuppression. Moreover, clodronate pre-treatment increased durable donor-specific BALB/c skin allograft tolerance. These results provide proof-of-principle that clodronate is effective at sparing the number of donor BM cells required to achieve durable hematopoietic chimerism and donor-specific skin allograft tolerance and justify further development of a tolerance protocol based on this principle.

Clinical application of vascularized composite allograft (VCA) transplantation has been limited and is controversial because of the high risk of toxicity from life-long immunosuppression relative to the benefit of acquiring a non-vital organ^1^,^2^,^3^,^4^. Therefore, a major goal in VCA, as well as in all other allotransplantation applications, is to identify safer and more effective protocols, such as induction of donor-specific tolerance^2^,^5^. In this regard, mixed donor hematopoietic chimerism induction has been demonstrated to promote donor-specific allograft tolerance^6^,^7^. We have previously reported successful induction of durable mixed chimerism and robust donor-specific tolerance in a mouse model by transplanting a vascularized bone allograft combined with transient immunosuppression^8^. However, attempts to achieve durable mixed hematopoietic chimerism and donor-specific skin allograft tolerance by transplanting allogeneic BM cells failed unless large doses of BM cells were infused^9^. Developing a method that would require lower, clinically feasible doses of donor BM cells and transient immunosuppression would have strong implications for developing tolerizing protocols potentially applicable in the clinic.

Despite significant breakthroughs in the field, attaining durable mixed hematopoietic chimerism with clinically feasible doses of donor BM generally requires aggressive recipient conditioning. Such regimens are thought to provide a competitive advantage to the infused donor BM cells, especially HSCs, by damaging (or destroying) endogenous stem cells, but are generally associated with significant morbidity and mortality^10^,^11^.

HSC activity in the niche is tightly controlled; however, under certain circumstances, they can be induced to egress from the BM and enter into the peripheral circulation, a process termed “mobilization”, which is still poorly understood^11^,^12^. A number of mobilizing agents and methods have been identified, such as the CXCR4 antagonist AMD3100 (plerixafor)^13^ and Granulocyte colony-stimulating factor (G-CSF)^14^, the VCAM-1/VLA-4 signaling antagonist BIO5192^15^, the chemokine CXCL2 (Groβ), Flt3 ligand (Flt3L) and stem cell factor (SCF)^16^, as well as BM macrophage depletion by liposomal clodronate^17^. Whether recipient HSC mobilization increases the number of “unoccupied” BM niches remains unclear, despite the fact that donor-derived HSC engraftment can be enhanced when recipient HSCs are mobilized^18^. Moreover, whether pharmacologic enhancement of donor HSC engraftment would be sufficient to induce durable mixed chimerism and donor-specific tolerance has never been addressed.

Previously, we have attempted unsuccessfully to enhance mixed chimerism induction and donor-specific tolerance by mobilizing recipient HSCs with AMD3100 and/or G-CSF before allogeneic BM infusion^9^. In the present study, we have attempted to mobilize recipient HSCs from BM niches by systemic depletion of recipient macrophages, and have found the approach effective in inducing durable mixed chimerism and robust donor-specific tolerance to skin allografts in recipient animals.

## Methods

### Mice

Male C57BL/6, BALB/c and CBA/Ca mice 8 to 12 weeks old (body weight range: 26 ~ 28 gram) were obtained from The Jackson Laboratory (Bar Harbor, Maine, USA) and were maintained in our specific pathogen-free animal facility. All care and handling of animals was carried out in accordance with guidelines provided in the Guide for the Care and Use of Laboratory Animals published by the U.S. Department of Health and Human Services. All studies were reviewed and approved by the Animal Care and Use Committee of the NIAID, NIH.

### Bone marrow and skin transplantation

Allogeneic BM transplantation was modified from a protocol described elsewhere^19^. Briefly, BALB/c BM cells were obtained by flushing long bones, and were suspended in complete medium containing Ammonium-Chloride-Potassium lysing buffer to remove red blood cells. Unseparated BM cells were injected i.v. into C57BL/6 recipient mice via tail vein. The number of cells injected was either 2 × 10^7^ or 5 × 10^7^, which are respectively below and above the threshold for establishing hematopoietic chimerism in our system without BM conditioning using radiation or cytotoxic agents. The immunosuppression protocol used in all experiments involved three consecutive 1 mg injections (days 0, 2 and 4 post- BM transplantation) of each of the following monoclonal antibodies: rat anti-mouse CD4 (CD4 cell non-depleting; YTS 177), anti-CD40 ligand (MR1) and anti-mouse CD8 (CD8 cell depleting; YTS 169), all from BioXcell (West Lebanon, NH). In addition, two doses of 12 mg/kg of rapamycin (LC Laboratories, Woburn, MA) were administered i.p. at days 6 and 30 post-transplantation^8^.

Where indicated, skin transplantation was performed as previously described by grafting full thickness tail skin measuring 1 × 1 cm on the two lateral flanks, one for the BALB/c donor type and the other for the CBA/Ca third party^8^. Grafts were observed daily after the removal of the bandage at day 7 post-transplantation and were considered rejected when complete loss of viable donor epithelium had occurred.

### *In vivo* macrophage depletion protocol

Our protocol of *in vivo* macrophage depletion was modified from that described previously^17^. Mice were retro-orbitally injected with 200 or 300 μL of a clodronate-loaded liposome suspension (Encapsula NanoSciences, Brentwood, TN) every other day for four doses during the week prior to BM transplantation and beginning immunosuppression. Control mice were injected with 300 μL PBS-loaded liposomes using the same schedule. Some mice received intra-nasal administration of liposomal clodronate to deplete lung macrophages; in that case, a total of 150 μL liposomal clodronate was administered on days one and two before BM transplantation^20^.

### Flow cytometry and analysis of chimerism

Leukocytes were purified from the blood, thymus, spleen, lymph node, BM, liver and lung from mice that had received BM transplantation, then were washed after red blood cell lysis and incubated for 20 min with Fc blocking antibody (Clone 93, 1:50 BioLegend, San Diego, CA) followed by the relevant antibody combinations (1:50 dilution) and FACS staining buffer for 30 min at 4 °C. Mixed chimerism was assessed in multiple lineages after staining cells with the following antibodies obtained from BioLegend or eBioscience (San Diego, CA): H-2K^d^ FITC or PB (SF1-1.1), CD4-APC-Cy7 or -PB (GK1.5), CD8-APC or -PE (53-6.7), CD19-APC-Cy7 (6D5), CD11b-FITC or eFluor 450 (M1/70), F4/80-APC or -PB (BM8). The recipient Vβ repertoire was screened with the following antibodies from BD Pharmingen (San Jose, CA): anti-Vβ5.1-5.2-PE (MR9-4), anti-Vβ6-PE (RR4-7), and anti-Vβ11-PE (RR3-15) in association with anti-CD4 and anti-CD8. All data were collected using an LSRII flow cytometer (BD Biosciences, San Jose, CA) and analyzed with FlowJo X software (version 10.0.7r2; Treestar, Ashland, OR).

### Immunofluorescence confocal imaging analysis

Immunofluorescence labeling and confocal imaging was performed using whole mount preparations of the sternum as previously described^21^. Sternums were bisected sagittally and were fixed with 4% paraformaldehyde at room temperature (RT) for 1 hr. After washing with PBS three times for 15 minutes, specimens were stained with rat anti-mouse CD169 (3D6.112, 1:50, AbD Serotec, Raleigh, NC) or anti-Nestin (rat-401, 1:50, EMD Millipore, Billerica, MA) antibodies followed by Texas-Red conjugated donkey anti-rat IgG (1:200, Jackson Immunoresearch, West Grove, PA). To track the donor cells engrafted in recipients, we stained BM with anti-mouse H-2K^d^-FITC (1:50, BioLegend). Finally, nuclei in the samples were counterstained with 4’, 6-diamidino-2-phenylindole (DAPI, 1:1000, Invitrogen, Carlsbad, CA) at RT for 40 minutes. All images were acquired directly under 10× NA 0.75 objectives using confocal light scanning microscopy on Zeiss LSM 510 and LSM 710 confocal systems (Confocal Zeiss MicroImaging, Jena, Germany). 3D tiled-images comprising the entire sternum fossae volume were collected for assessment of the number and distribution of donor and recipient cells.

### Statistics

Data were analyzed using unpaired parametric *t*-tests (two-tailed) with Prism 6 (GraphPad Software) and are presented as the mean ± SEM of summary data (the data were approximately normally distributed).

## Results

### Clodronate pretreatment induces durable mixed hematopoietic chimerism associated with robust, donor-specific skin allograft tolerance

The use of liposomal clodronate in mice, especially frequent and large intravenous doses, is known to be toxic and even fatal in a subset of animals^22^. We confirmed this in a preliminary experiment, in which we tested 100, 200 and 300 μL doses of liposomal clodronate given IV every other day for a total of four doses (n = 5 in each group). We observed one, two and two deaths per group, respectively (overall mortality rate of 33.3%), occurring between days 2 and 14 after the first injection; no deaths were observed in a group receiving control liposomes (n = 5) (data not shown). Since the majority of animals survived, we decided this was sufficient to test the concept that this macrophage-depleting agent could enhance mixed chimerism and induce donor-specific tolerance. Consistent with our preliminary results, we found an overall mortality rate of 31.8% across all the experiments detailed below (14 deaths in 44 mice receiving IV liposomal clodronate). Stratified by the doses studied, we found that 5 out of 15 mice (33.3%) receiving 200 μL of liposomal clodronate died, and 9 out of 29 mice (31.03%) receiving 300 μL of liposomal clodronate died, whereas no deaths were observed in mice receiving intranasal liposomal clodronate (n = 6), i.v. control liposomes (n = 12) or no liposomes (n = 13) (Table 1). With one exception, all mice receiving liposomal clodronate that survived this early phase of high mortality survived until the end of the study (>180 days after clodronate administration) and were not distinguishable from control mice with regard to weight and overt behavior. The outlier mouse had received 200 μl of liposomal clodronate and died on day 62 after the first injection.

Using the same dosing schedule specified above, we found that injection of 300 μl of liposomal clodronate resulted in a 50% reduction of CD11b^+^ F4/80^+^ macrophages in the BM compared to control liposome-injected animals on the day after the last injection (p < 0.001, n = 2 experiments), consistent with previously published studies^17^. No effect was observed for liposomal clodronate on the absolute levels of neutrophils, B cells or T cells in the blood (data not shown).

**Figure 1:**
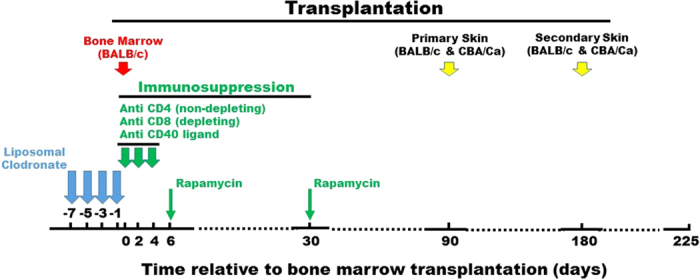
Experimental protocol for liposomal clodronate enhancement of hematopoietic chimerism and donor-specific skin allograft tolerance. Recipient C57BL/6 mice were pretreated i.v. with liposomal clodronate or control liposomes at the times indicated by the blue arrows before the day of allogeneic BM transplantation (day 0). Immunosuppressive agents were then given where indicated by the green arrows (see Materials and Methods for doses and details). Donor-specific tolerance was tested by applying both BALB/c and CBA/Ca skin allografts on the dorsum of recipient mice at both 90 days (primary allografts) and 180 days (secondary allografts) after BM transplantation. Animals were sacrificed on day 225 post BM transplantation.

On the day after the last liposomal clodronate or control liposome injection of C57BL/6 recipient mice, we infused allogeneic BALB/c donor BM to test whether mixed chimerism induction could be enhanced under transient immunosuppression conditions (administration on days 0, 2 and 4 post-BMT of depleting anti-CD8 mAb and non-depleting anti-CD4 mAb and CD40L mAb; rapamicin on day 6 and day 30 post-BMT) (Fig. 1). Infusion of 20 million donor BM cells into recipient mice receiving control liposomes induced only a background level of mixed chimerism in peripheral blood, ranging from 0.05 ± 0.02% to 0.14 ± 0.07%, both at 30 and 90 days after transplantation. In contrast, in mice receiving 50 million BM cells and the same immunosuppression schedule, without any clodronate pretreatment, durable mixed chimerism was established (0.86 ± 0.21% and 2.01 ± 0.66% at 30 and 90 days, respectively) (Supplemental Fig. 1A). These benchmarks are consistent with our previous report^9^.

C57BL/6 mice receiving 20 million BALB/c donor BM cells (i.e., the dose we demonstrated to be below a threshold required to establish hematopoietic chimerism under transient immunosuppression) showed clear evidence of durable mixed hematopoietic chimerism when they were pre-treated with four every other day injections of 200 μl of liposomal clodronate before receiving the transplant, in contrast to mice receiving the same doses of control liposomes and BM cells (Supplemental Fig. 1A).

Skin allotransplantation with donor-type (BALB/c) and third-party (CBA/Ca) allografts was performed next in these chimeric mice to determine whether donor-specific skin allograft tolerance could be established in association with clodronate-dependent hematopoietic chimerism. Skin allotransplantation was first performed (primary allograft) 90 days after the BM transplant, a time-point when all the recipient mice had been off immunosuppression for at least two months. As expected, all third-party CBA/Ca primary allografts were rapidly rejected (fewer than 20 days) after skin transplantation in all groups tested. The BALB/c donor-type primary allografts were also rapidly rejected in mice receiving 20 million donor BM cells without liposomal clodronate pretreatment. In contrast, prolonged donor-specific primary skin allograft survival was observed in mice pretreated with liposomal clodronate. In particular, in liposomal clodronate-pretreated mice, rejection was markedly delayed in 3 out of 7 animals; in the other 4 mice, no evidence of rejection was observed for up to 90 days post-primary skin allotransplantation (Supplemental Fig. 1B), at which point the animals were sacrificed to evaluate immune organ chimerism (see below).

**Figure 2:**
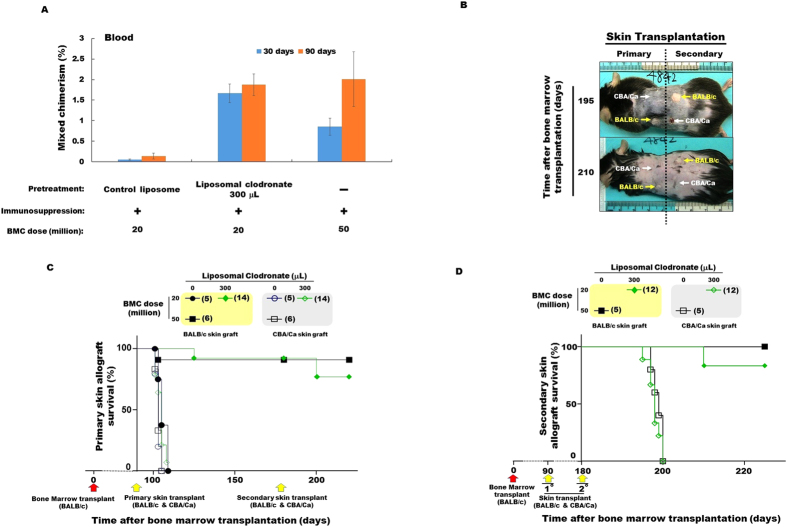
Liposomal clodronate pretreatment enhances mixed hematopoietic chimerism and donor-specific skin allograft tolerance. Mice receiving BM transplantation were subjected to both BALB/c and CBA/Ca skin transplantation both 90 days (primary allografts) and 180 days (secondary allografts) later, as indicated in Fig. 1. (**A**) Clodronate-enhanced mixed hematopoietic chimerism. The percentage of cells expressing BALB/c MHC-class I (H-2K^d^) in peripheral blood, expressed as mean ± SEM, was checked at the time-points after BM transplantation coded at the upper right. (**B**) Donor-specific tolerance. The same representative recipient mouse with all four skin allografts in place (two primary and two secondary to the left & right of the dotted line, respectively) is shown at the time points indicated to the left of each image. Arrows indicate the position of each skin transplant. (**C,D**) Quantitation of donor-specific tolerance for the primary (**C**) and secondary (**D**) skin allografts. The symbol code defining the number of transplanted BM cells and the dose of liposomal clodronate in each skin graft type is shown in the boxes above panels C and D; the number of mice in each group is given in the parentheses next to each symbol.

We next carried out a second independent set of experiments, this time increasing the dose of liposomal clodronate to 300 μL per injection. Mixed hematopoietic chimerism was also observed in these animals, 1.67 ± 0.22% and 1.87 ± 0.26% at 30 and 90 days, respectively (Fig. 2A), which are similar to the levels of chimerism we had observed using 200 μL per injection of liposomal clodronate (Supplemental Fig. 1A). Again, animals receiving control liposomes had only background levels of chimerism, and animals receiving 50 million BM cells without liposomes had durable and high levels of chimerism, similar in magnitude to what was observed with clodronate-pretreated mice receiving a subthreshold dose of 20 million BM cells (Fig. 2A). Furthermore, only 2 out of 14 mice receiving 300 μL of liposomal clodronate per injection underwent donor-type BALB/c skin allograft rejection during the observation period post primary skin allotransplantation (Fig. 2B,C). The other twelve donor-type primary BALB/c allografts survived for the full post-primary skin transplant observation period (90 days). Rejection of the donor-type BALB/c primary skin allografts in the two exceptions was markedly delayed (35 days post-primary skin transplantation) compared to the time of the primary third party CBA/Ca allograft rejection (which occurred in 100% of mice), as well as compared to the time of donor type primary allograft rejection in mice not receiving liposomal clodronate. This rate of donor-type BALB/c primary skin allograft survival was similar to the survival rate in C57BL/6 mice receiving 5 × 10^7^ donor BM cells (cells) without liposomal clodronate pretreatment (Fig. 2B,C).

To test the robustness of the apparent donor-specific primary skin allograft tolerance in these 12 mice that had received 300 μL of liposomal clodronate per injection, donor-type and third party secondary skin allotransplantation was performed. Ten of the 12 mice tolerated both the primary and secondary donor-type BALB/c allografts for the full observation period after secondary transplantation (45 days), whereas all third party CBA/Ca secondary skin allografts were rejected in fewer than 20 days. Rejection of primary and secondary donor-type allografts in the other two mice occurred 20 and 30 days after the time of secondary skin transplantation (Fig. 2B,D).

### Multi-lineage chimerism was found in tolerant animals, with donor-derived B lymphocytes the major component

**Figure 3:**
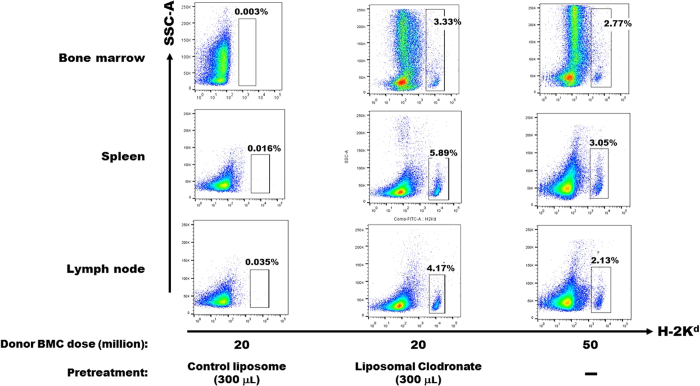
Clodronate-induced chimerism is durable in immune organs. The immune organs indicated to the left of each row were removed on day 225 post BM transplantation of the groups indicated at the bottom of each column, corresponding to the mice analyzed in Fig. 2, and hematopoietic chimerism was assessed by flow cytometry. The data shown are from one mouse from each of the treatment groups defined at the bottom of each column of plots and are representative of a total of 5 (control liposomes), 10 (clodronate) and 5 (50 million cells, no clodronate) mice analyzed from each group combined from 2 independent experiments performed.

Mice in the low dose clodronate group (200 μL/injection) were sacrificed at 180 days after BM infusion (i.e. 90 days after primary skin allograft transplantation) to check the status of mixed chimerism in immune organs (spleen, lymph node, BM) as well as in liver, lung and kidney. Only background levels of hematopoietic chimerism were observed in mice that had received 20 million donor BM cells without liposomal clodronate pretreatment (Supplemental Fig. 1C). In contrast, in the liposomal clodronate-pretreated mice receiving 20 million BM cells, 3–6% mixed hematopoietic chimerism was observed in immune organs of the mice still tolerant to donor-type skin allografts (Supplemental Fig. 1C, and data not shown). Mixed chimerism was also observed in the liver and lungs of the clodronate-pretreated mice; no chimerism was observed in the kidney (data not shown). On the other hand, in the few clodronate pretreated mice that had rejected their skin allografts, mixed chimerism did not rise above the background level (data not shown). In those mice receiving 50 million donor BM cells without clodronate pre-treatment, 2–4% mixed chimerism was also observed in immune organs of mice remaining tolerant to donor-type skin allografts (Supplemental Fig. 1C). The same pattern of mixed hematopoietic chimerism was observed in the same organs for the high dose liposomal clodronate-treated group of mice described above (300 μL/injection) (Fig. 3). It is important to note that since the high dose but not the low dose clodronate group of mice was subjected to secondary skin allotransplantation, mice in this group were sacrificed even later than the low dose group (at 225 days, instead of 180 days as for the low dose group, after BM infusion; i.e. 135 days after primary skin allograft transplantation) (Fig. 3).

**Figure 4:**
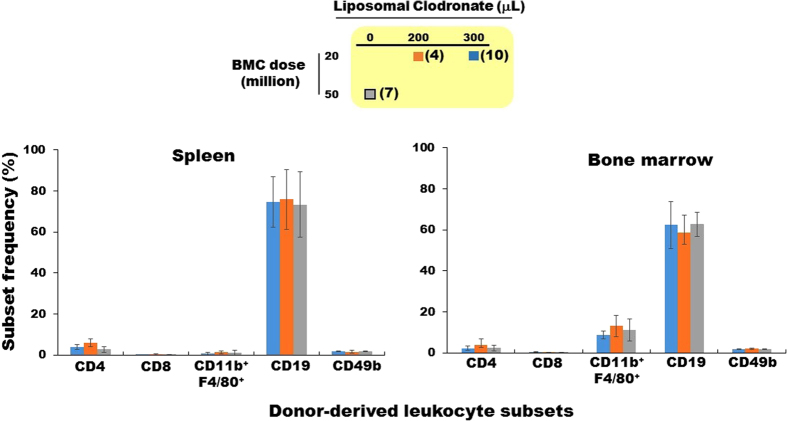
Donor-derived cells in tolerant mice are predominantly B lymphocytes. The percentage of cells expressing BALB/c MHC-class I (H-2K^d^) in spleen and BM from recipient mice, expressed as mean ± SEM, was determined at the end of all experiments (180 and 225 days post BM transplantation for mice receiving 200 versus 300 μl of liposomal clodronate, respectively). The code for the number of BM cells and the dose of liposomal clodronate for each treatment group is in the box at the top of the figure. Results are summarized from two independent experiments with a total of 4–10 mice in each group (specified in parentheses in the symbol code box).

**Figure 5:**
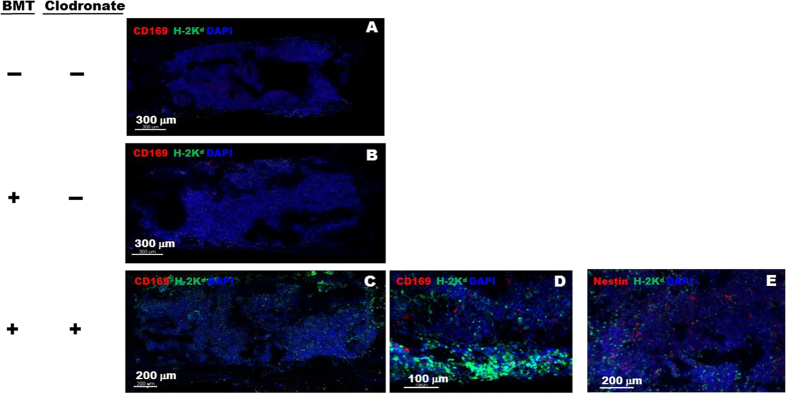
Imaging of clodronate enhanced donor hematopoietic cell engraftment in BM, including cells proximal to stem cell niches. (**A**) BM image of normal (strain) control mice without BM transplantation (BMT) and clodronate treatment. (**B**) BM image of C57BL/6 mice that received BMT from BALB/c mice, but without clodronate treatment. (**C–E**) BM images of C57BL/6 mice pretreated with clodronate followed by BMT from BALB/c donor mice. Images were acquired by confocal microscopy at the time of sacrifice (day 225 after BMT). Nuclei (blue, DAPI), BALB/c MHC-class I molecule H-2K^d^ (green, FITC), CD169^+^ macrophages (red, Texas-Red) or Nestin^+^ mesenchymal stromal cells (red, Texas-Red). Scale bars are in micrometers. The images shown are representatives from the same two independent experiments detailed in Fig. 2 (total n = 3 ~ 5 mice per group).

Lineage analysis of donor-derived cells in the BMs of recipient mice treated with either high or low dose clodronate revealed that CD19-positive B lymphocytes predominated, followed by CD11b^+^ F4/80^+^ macrophages, CD4^+^ T lymphocytes, and CD49b^+^ NK cells (Fig. 4). Histological analysis of chimeric recipient mice revealed the presence of donor MHC class I-expressing cells *in situ* in the BM, mainly in the peripheral sub-endosteal space (Fig. 5). Some of these donor-derived cells were found adjacent to cells expressing Nestin, a marker of mesenchymal stromal cells in BM niches (Fig. 5E). Both findings suggested that engraftment of the donor-derived cells had occurred in the recipient BM stem cell niche.

### Donor-specific skin allograft tolerance is associated with specific Vβ T-cell deletion

**Figure 6:**
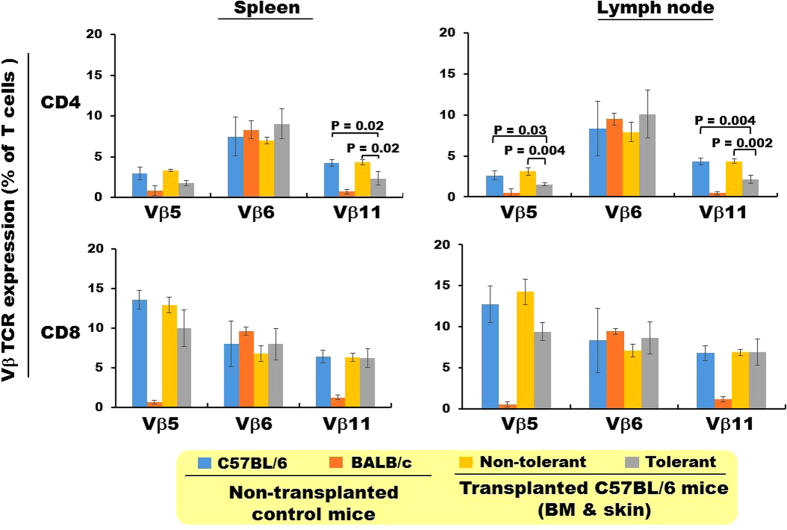
Specific Vβ-expressing T cell deletion occurs in mice exhibiting donor-specific tolerance. The frequency of specific Vβ−expressing T cells was measured at day 225 post BM transplantation for both CD4^+^ and CD8^+^ T cells in both spleen and lymph node for the four groups of mice defined in the box below the graph. Results are expressed as mean ± SEM and are summarized from the two independent experiments detailed in both Fig. 2 and Supplemental Figure 1 (total n = 3–6 mice per group as detailed in the parentheses next to each symbol in the symbol code box). Statistically significant differences are shown by the p values.

One of the mechanisms by which mixed donor-recipient chimerism is thought to promote donor-specific tolerance is by central (intrathymic) and peripheral deletion of donor-reactive T cells from the recipient^23^. In our transplantation model, this can be reflected by the degree of deletion of specific Vβ T cells^8^. In particular, only the BALB/c MHC class II molecules present superantigen to TCR bearing Vβ5 and Vβ11 subdomains, leading to deletion of T cells expressing these Vβ molecules^8^. Thus, we tested whether specific Vβ T cell deletion was present in our model by comparing the percentages of Vβ5 and Vβ11 T cells in tolerant and non-tolerant skin allograft recipients as well as in naive animals; Vβ6-expressing T cells, which do not recognize endogenous superantigens, served as control. The percentages of Vβ6-expressing CD4^+^ and CD8^+^ T cells did not differ among the groups. In contrast, a significant reduction of Vβ11-expressing CD4^+^ T cells was observed in the spleen and lymph node of mice tolerant to the skin allografts compared to the other two groups (Fig. 6). A similar decrease was also observed in the Vβ5-expressing CD4^+^ T cells in the lymph node. No difference was observed for CD8^+^ T cells.

### Systemic administration of liposomal clodronate and transient immunosuppression are critical for durable mixed chimerism induction

**Figure 7:**
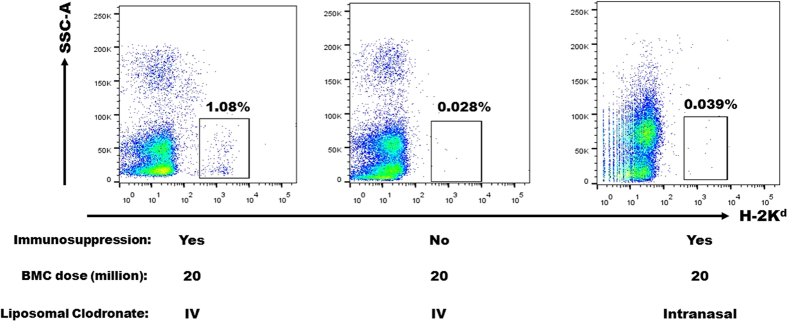
Enhancement of mixed chimerism does not occur in the absence of transient immunosuppression or if liposomal clodronate is given intranasally. Peripheral blood mixed chimerism, defined by the percentage of BALB/c MHC-class I (H-2K^d^)-positive cells, was determined at 30 days post-BM transplantation in recipient C57BL/6 mice under the conditions specified below each plot. Mice did not receive skin transplantation. Data are representative of two independent experiments with the same pattern (total n = 6 in each group).

A possible mechanism underlying our finding is that depletion of macrophages in the lungs decreased the passive entrapment of infused donor BM cells (first-pass effect), thus increasing the probability of BM niche engraftment. To test this, we performed BM infusion after administering liposomal clodronate via the intranasal route, a classical approach to selectively deplete lung macrophages^20^. In a control group of mice, BM infusion was performed after systemic macrophage depletion but in the absence of any immunosuppression. In contrast to mice receiving both systemic macrophage depletion and transient immunosuppression, no mixed chimerism was observed 30 days after BM infusion in either mice receiving intranasal clodronate or mice not receiving immunosuppression, suggesting the importance of systemic macrophage depletion and transient immunosuppression in the induction of mixed chimerism (Fig. 7).

## Discussion

In the present study, we have demonstrated that the macrophage-depleting agent clodronate promotes durable mixed hematopoietic chimerism and donor-specific skin allograft tolerance in mice. It has been known since the 1940’s that a state of mixed hematopoietic chimerism in allotransplant recipients relative to the genetic background of the donor may be associated with donor-specific tolerance^24^. Despite the clinical promise of donor-specific tolerance and the reality that it can be achieved by establishing mixed hematopoietic chimerism under specific laboratory conditions and in rare experiments of nature, the ability to translate knowledge in this area to a safe and effective treatment for patients has been limited not only by the need for harsh patient conditioning but also by the requirement for clinically unachievable doses of BM cells needed to establish chimerism. Any protocol that might reduce the BM cell dose requirement would represent a significant advance in this field. We have previously described a relatively transient immunosuppressive regimen in mice that supports establishment of hematopoietic chimerism in the absence of radiation and cytotoxic agents, but this regimen still requires 50 million BM cells from the allogeneic donor or else transplantation of an intact limb^8^,^9^. Chimerism is thought to involve engraftment of donor hematopoietic stem cells in BM niches. The probability of donor HSC engraftment is thought to depend in part on the number of niche vacancies in the recipient, which may be increased by targeting specific niche components with specific drugs. The idea is to transiently create space in the niches at the time of BM transplantation to allow allogeneic HSCs to enter and engraft. In this regard, we have previously tested plerixafor, an antagonist of the HSC retention factor and chemokine receptor CXCR4, and found that it could enhance hematopoietic chimerism after BM transplantation, but only transiently and without promoting donor-specific allograft tolerance^9^.

Several studies have demonstrated that monocyte/macrophage depletion may promote mobilization of HSCs from BM to peripheral blood and spleen in mice^17^,^25^,^26^. The mechanism is thought to involve loss of direct support of niche cell HSC retention activity by local BM macrophages, although other potential mechanisms may operate. Importantly, in animals pretreated with clodronate durable mixed chimerism occurred after infusing only 20 million total donor BM cells under transient immunosuppression conditions, and was associated with robust, donor-specific skin allograft tolerance. This was evidenced by the selective rejection of third-party but not donor-type fresh skin allografts transplanted 90 & 180 days after donor BM infusion, time-points at which the mice were free of immunosuppression. Our results using liposomal clodronate suggest that niches are fully competent after macrophage depletion in supporting allogeneic HSC engraftment, self-renewal and differentiation, as evidenced by the durable multilineage mixed chimerism and specific donor type Vβ T cell depletion we observed in chimeric recipient animals.

A number of other mixed chimerism approaches have been investigated trying to transplant clinically realistic doses of BM without irradiation or cytotoxic drugs^24^. Among them, only infusion of *in vitro*-prepared, polyclonal recipient-type regulatory T cells (Tregs) leads to engraftment of clinically feasible doses of fully allogeneic BM without cytoreductive recipient conditioning^27^. In that study, although long-term, donor-specific skin allograft tolerance was achieved, the robustness of tolerance was not determined by second-set grafting of fresh skin. In any case, preparing Tregs *ex vivo* is complicated and expensive and may not scale as a community-based treatment option.

Our study has several important limitations. First, although it did deplete macrophages in the BM and in other organs, we do not know exactly how clodronate is working. Second, clodronate was toxic and resulted in ~30% mortality in recipient mice, mainly due to cardiac complications (infarction and hemorrhage). We took advantage of the surviving mice, which represented the majority, to perform this proof-of-principle study; however, a role for one or more of the toxic side-effects of clodronate at the doses we used cannot be excluded with regard to promoting chimerism. Third, our data indicate that immunosuppression, although transient, is still needed to induce a state of mixed hematopoietic chimerism and possibly to prevent graft-versus-host disease (GVHD).

In conclusion, we have developed proof-of-principle that pretreatment of recipient mice with the macrophage-depleting agent liposomal clodronate can enhance induction of mixed hematopoietic chimerism and promote robust donor-specific skin allograft tolerance. In order to be translated to the clinic, further optimization of the protocol will be necessary, at the level of both safety and efficacy, or instead by identifying another less toxic macrophage-depleting agent able to enhance chimerism and tolerance.

## Additional Information

**How to cite this article**: Li, Z. *et al.* The Macrophage-depleting Agent Clodronate Promotes Durable Hematopoietic Chimerism and Donor-specific Skin Allograft Tolerance in Mice. *Sci. Rep.* **6**, 22143; doi: 10.1038/srep22143 (2016).

## Supplementary Material

Supplementary Information

## Figures and Tables

**Table 1:** Survival in mice receiving liposomal clodronate.

Treatments			Numbers			
Control liposome	Liposomal Clodronate	Immunosuppression	BMT	Death	Survival	Total
No	Yes (200 μL IV)	Yes	Yes	5	10	15
No	Yes (300 μL IV)	Yes	Yes	6	14	20
No	Yes (300 μL IV)	No	Yes	3	6	9
			Total:	14	30	44
No	Yes (150 μL IN)	Yes	Yes	0	6	6
Yes (200 μL IV)	No	Yes	Yes	0	7	7
Yes (300 μL IV)	No	Yes	Yes	0	5	5
No	No	Yes	Yes	0	13	13
			Total:	0	25	25

Abbreviations: BMT, bone marrow transplantation; IN: intranasal.
