# Supplementary material for: The Macrophage-depleting Agent Clodronate Promotes Durable Hematopoietic Chimerism and Donor-specific Skin Allograft Tolerance in Mice
Source: Sci Rep. 2016 Feb 26;6:22143. doi: 10.1038/srep22143 (PMC4768260; doi:10.1038/srep22143)
Supplement: Supplementary Information [file srep22143-s1.pdf]

The Macrophage-depleting Agent Clodronate Promotes Durable Hematopoietic Chimerism and Donor-specific Skin Allograft Tolerance in Mice

Zhanzhuo Li[1], Xin Xu[1], Xingmin Feng[2] and Philip M. Murphy[1]

[1]Laboratory of Molecular Immunology, National Institute of Allergy and Infectious Diseases (NIAID), National Institutes of Health, Bethesda, MD, USA

[2]Hematology Branch, National Heart, Lung and Blood Institute (NHLBI), National Institutes of Health, Bethesda, MD, USA

Correspondence: Philip M. Murphy, M. D., Bldg 10, Room 11N113, NIH, Bethesda, MD 20892; T: 301-496-8616; F: 301-402-4369; e: pmm@nih.gov

A

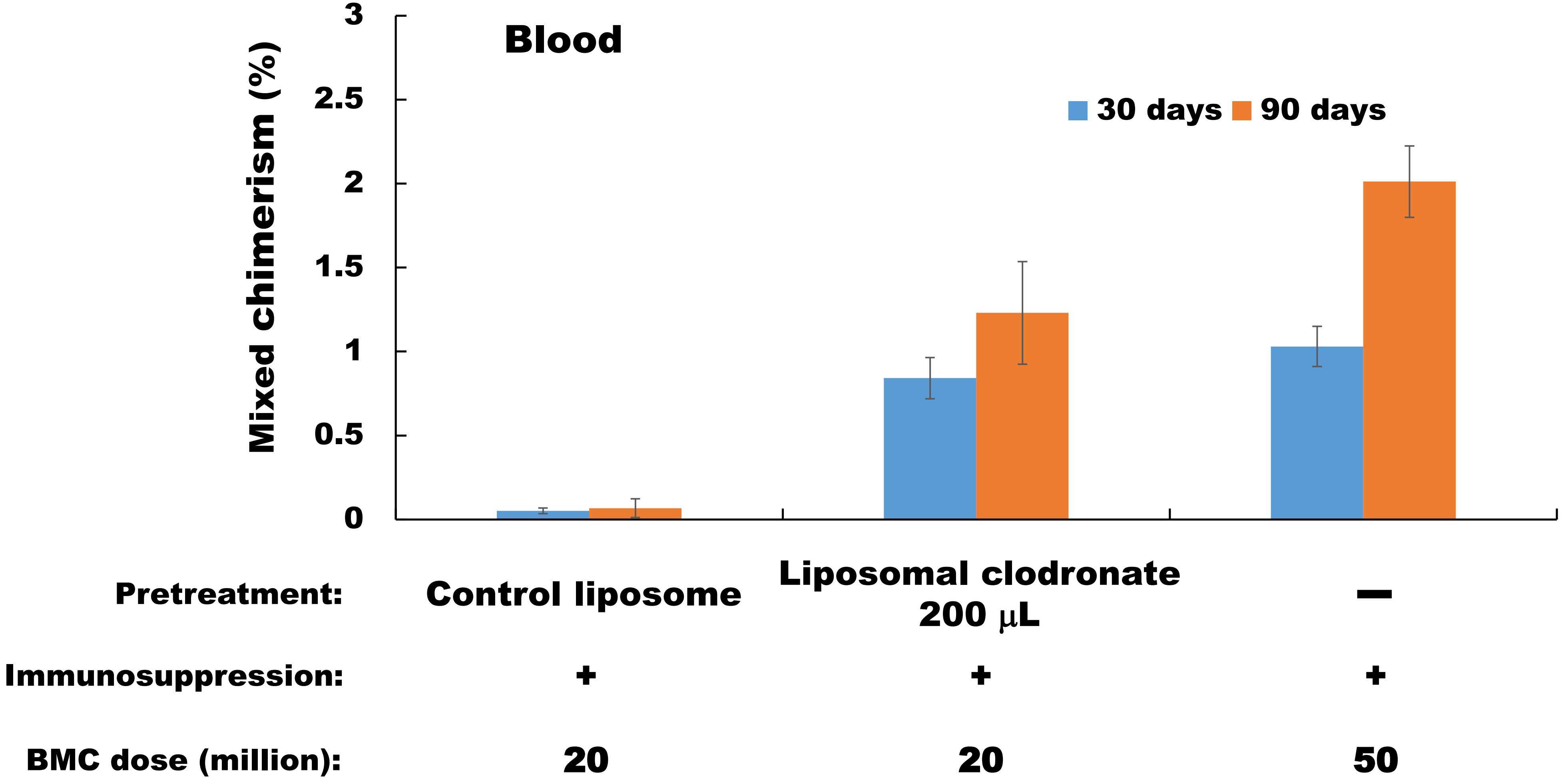
Blood
Mixed chimerism (%)
3
2.5
2
1.5
1
0.5
0
30 days
90 days
Pretreatment:
Control liposome
Liposomal clodronate 200 μL
—
Immunosuppression:
+
+
+
BMC dose (million):
20
20
50

B

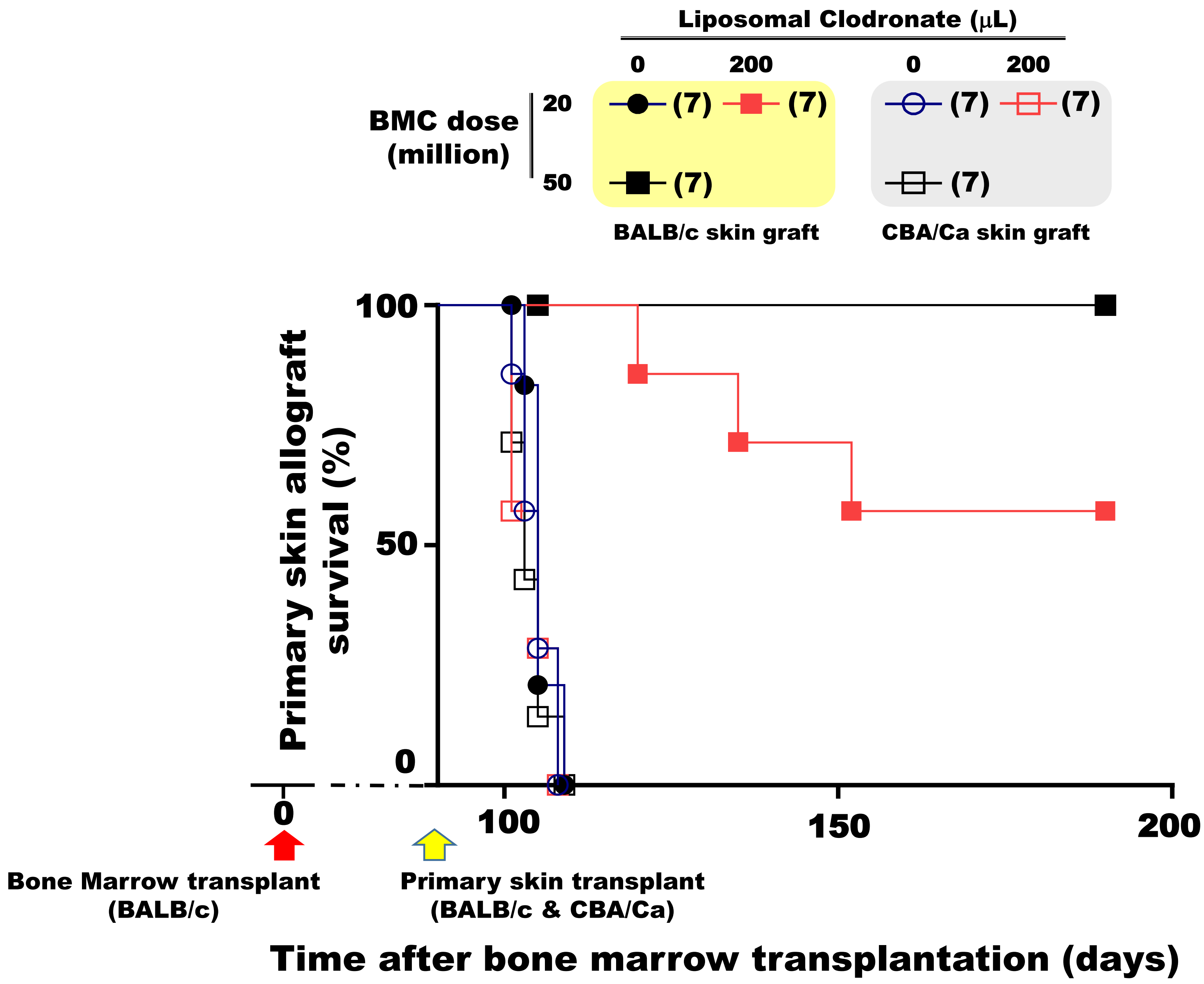
Liposomal Clodronate (μL)
0
200
0
200
BMC dose
(million)
20
50
(7)
(7)
(7)
(7)
(7)
(7)
BALB/c skin graft
CBA/Ca skin graft
Primary skin allograft survival (%)
100
50
0
0
100
150
200
Bone Marrow transplant
(BALB/c)
Primary skin transplant
(BALB/c & CBA/Ca)
Time after bone marrow transplantation (days)

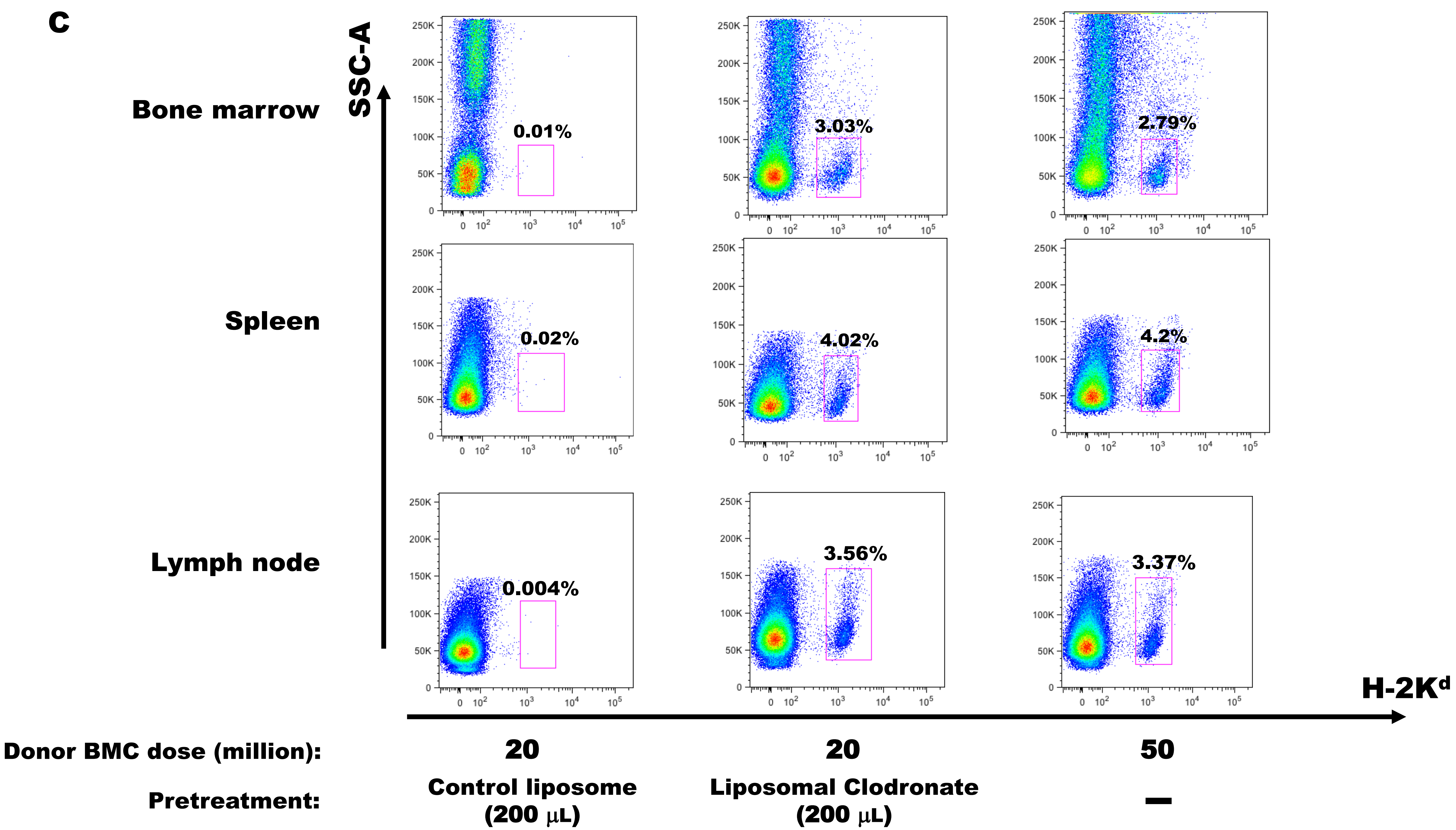
c
SSC-A
H-2Kd
Bone marrow
Spleen
Lymph node
0.01%
3.03%
2.79%
0.02%
4.02%
4.2%
0.004%
3.56%
3.37%
Donor BMC dose (million):
20
20
50
Pretreatment:
Control liposome (200 μL)
Liposomal Clodronate (200 μL)
—

Supplemental Figure Legends

Supplementary Figure 1: Low dose liposomal clodronate (200 μl) pretreatment enhances mixed hematopoietic chimerism and donor-specific skin allograft tolerance. A) Enhanced mixed hematopoietic chimerism. The percentage of cells expressing BALB/c MHC-class I in peripheral blood ($H\text{-}2K^d$), expressed as mean ± SEM, was checked at the time-points indicated at the upper right after bone marrow transplantation under the conditions specified below the x-axis. B) Donor-specific skin allograft tolerance. C57BL/6 mice receiving BALB/c bone marrow transplantation were subjected to both BALB/c (donor type, filled marks) and CBA/Ca (third party, open marks) primary skin transplantation 90 days later, as indicated by the arrow. The symbol code is shown in the box above the figure with the number of mice in each group in parentheses next to each symbol. Primary skin allograft survival is shown. C) Clodronate-induced chimerism is durable in immune organs. The immune organs indicated to the left of each row of the mice defined below each column of plots were analyzed on day 180 post bone marrow transplantation for hematopoietic chimerism as assessed by flow cytometry. The data shown are from one mouse from each of the treatment groups and are representative of a total of 7 (control liposomes), 4 (clodronate) and 7 (50 million cells, no clodronate) mice analyzed from each group combined from the 2 independent experiments performed.
